# NanoSTR: A method for detection of target short tandem repeats based on nanopore sequencing data

**DOI:** 10.3389/fmolb.2023.1093519

**Published:** 2023-01-18

**Authors:** Jidong Lang, Zhihua Xu, Yue Wang, Jiguo Sun, Zhi Yang

**Affiliations:** Department of Bioinformatics and Application Development, Qitan Technology Co., Ltd., Beijing, China

**Keywords:** nanopore sequencing, long read sequencing, short tandem repeat, STR, NanoSTR

## Abstract

Short tandem repeats (STRs) are widely present in the human genome. Studies have confirmed that STRs are associated with more than 30 diseases, and they have also been used in forensic identification and paternity testing. However, there are few methods for STR detection based on nanopore sequencing due to the challenges posed by the sequencing principles and the data characteristics of nanopore sequencing. We developed NanoSTR for detection of target STR loci based on the length-number-rank (LNR) information of reads. NanoSTR can be used for STR detection and genotyping based on long-read data from nanopore sequencing with improved accuracy and efficiency compared with other existing methods, such as Tandem-Genotypes and TRiCoLOR. NanoSTR showed 100% concordance with the expected genotypes using error-free simulated data, and also achieved >85% concordance using the standard samples (containing autosomal and Y-chromosomal loci) with MinION sequencing platform, respectively. NanoSTR showed high performance for detection of target STR markers. Although NanoSTR needs further optimization and development, it is useful as an analytical method for the detection of STR loci by nanopore sequencing. This method adds to the toolbox for nanopore-based STR analysis and expands the applications of nanopore sequencing in scientific research and clinical scenarios. The main code and the data are available at https://github.com/langjidong/NanoSTR.

## Introduction

Short tandem repeats (STRs), also known as microsatellites, are repetitive DNA sequences consisting of 1–6-bp motifs present in a genome. These highly individual-specific number of repeats and the abundance of motifs have contributed to the polymorphism of STR loci (Edwards et al., 1991). On average, STR loci occur every 15 kb in the human genome (Lander et al., 2001; Collins et al., 2003; Ellegren, 2004; de Koning et al., 2011). The number of repeat units differs between individuals, resulting in highly complex allele polymorphisms. Because of their high diversity, wide distribution, and high polymorphism, STRs are considered as the second generation of genetic markers after restriction fragment length polymorphisms (RFLP). Therefore, STR detection has been widely used in forensic identification, paternity testing, species polymorphism identification, and genetic disease diagnosis (La Spada et al., 1992; A novel gene containing a, 1993; Kayser, 2017; Alonso et al., 2018). Studies have shown that STRs represent a source of phenotypic variations in more than 30 Mendelian diseases, such as neurological disorders (Tang et al., 2017; Paulson, 2018).

Nanopore sequencing is an evolving third/fourth generation sequencing technology for direct detection of nucleotide sequences with kb or even Mb base pairs (Magi et al., 2018; Wang et al., 2021). In practice, however, the high error rate and special data characteristics of long-read sequencing have limited the efficient identification of STR polymorphisms, and therefore, further evaluation of the analytical methods is required (Magi et al., 2017; Rang et al., 2018). There are a few methods for STR identification based on nanopore sequencing, and the representative software are Tandem-Genotypes (Mitsuhashi et al., 2019), NanoSatellite (De Roeck et al., 2019), STRique (Giesselmann et al., 2019), etc. These software and related algorithms have limitations and shortcomings. For example, NanoSatellite directly analyzes STRs based on electric current distribution, and the accuracy of analysis depends heavily on the stability of the sequencing current and the precision of the basecalling model. Tandem-Genotypes requires data preprocessing steps such as LAST alignment and establishment of a genomic background database, and histograms are needed to assist STR genotyping. Therefore, the whole process is time-consuming. Other analytical methods such as NCRF (Harris et al., 2019) and TideHunter (Gao et al., 2019) are incapable of STR typing. Therefore, these analytical methods have limited applications and insufficient robustness.

**FIGURE 1:**
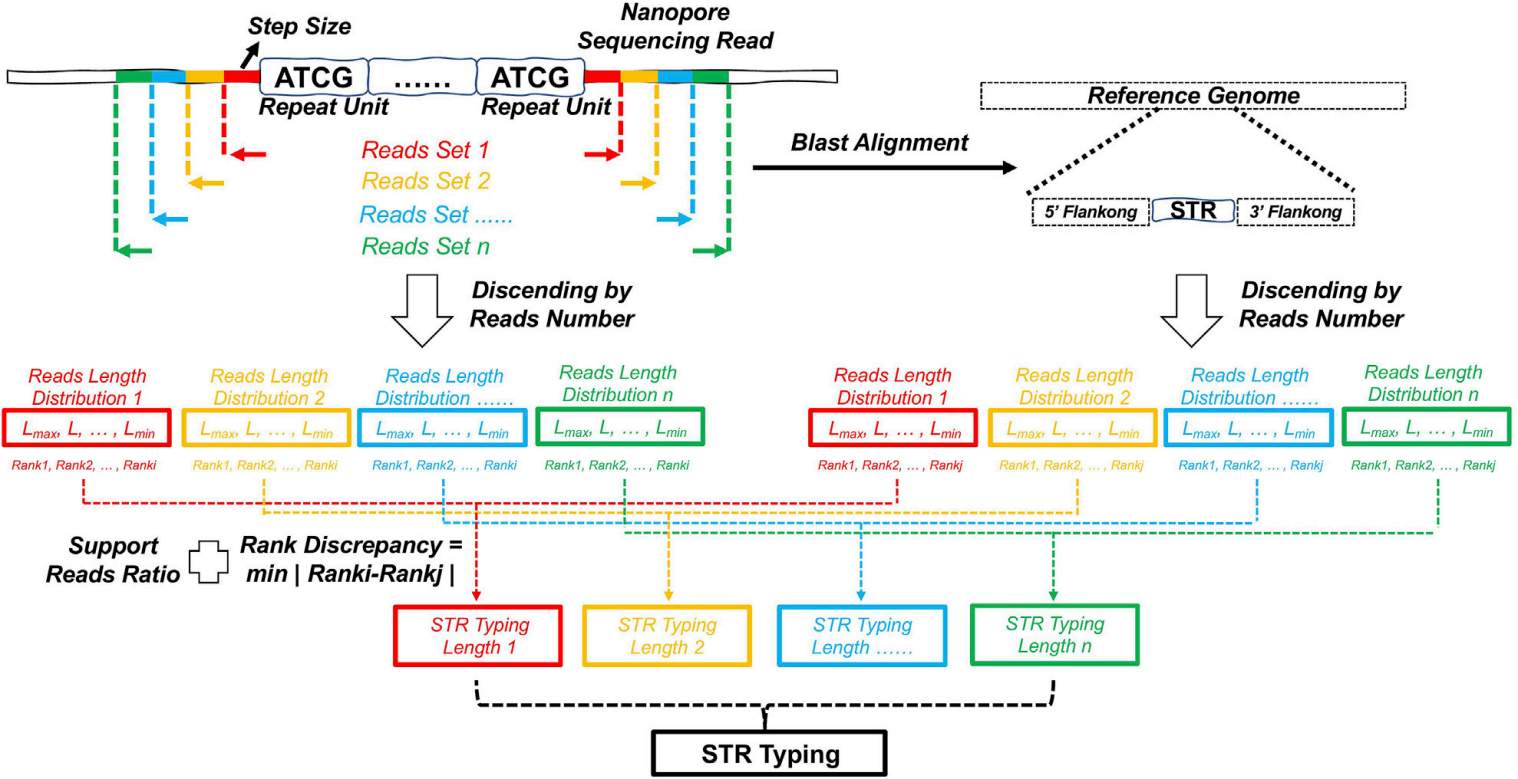
Schematic diagram of STR typing with NanoSTR. NanoSTR can be used to genotype STR loci based on multisampling and length-number-rank (LNR) information of reads. And the multiple genotypes are combined for statistical analysis, and the results with the mode and supported read number are selected as the final genotype for this target STR locus. After that, a secondary correction is performed according to the difference in the order of magnitude of the number of reads.

We therefore developed NanoSTR as a method for detecting target STRs based on nanopore sequencing. The method uses statistical analysis methods such as multisampling and the length-number-rank (LNR) information of reads for the genotyping and correction of STR markers with improved accuracy (Figure 1). In terms of data characteristics, NanoSTR effectively avoids the non-random sequencing errors and unexpected insertions-deletions (indels) associated with nanopore sequencing (Magi et al., 2017; Wang et al., 2021) and thus improves the efficiency of sequencing data utilization, the detection rate of STR genotypes, and the accuracy of STR profiling.

## Materials and methods

### Analysis principles and usage

Analysis with NanoSTR comprises the following four steps (Figure 1). The first step is definition of the extension step size d. The start and end positions of the target STR locus on the reference genome are marked as P_start and P_end. Extension is repeated N times to the upstream of P_start and to the downstream of P_end. The P_start’ and P_end’ of each extension are expressed as follows:

P_start_i_’ = P_start − d*i.

P_end_i_’ = P_end + d*i.

Where 1 ≤ i ≤ N.

The sequences with P_start_i_’ as the start position, P_end_i_’ as the end position, and d as the extension step size were extracted from the reference genome, which are referred to as paired-seed sequences. The N paired-seed sequences obtained after N extensions are used for the extraction of the complete matching target sequences from the nanopore sequencing data in *.fastq format to yield N datasets of target sequences. Then, the lengths of the target sequences in each dataset are determined to generate N datasets containing the sequence lengths. Finally, the lengths of the target sequences in each dataset are sorted in descending order of supported read number, and the sorted lengths are numbered in ascending order, which is defined as “rank.” Consequently, dataset1 with N subsets containing the length-number-rank (LNR) information of sequences is generated. In the second step, the target STR loci are extended over a certain distance (e.g., 500 bp by default) upstream of the start position and downstream of the end position on the reference genome, which are used as the reference sequences. Then, the N datasets of the target sequences obtained in the first step are aligned against the reference sequences using BLAST. The results in *m8* format are filtered with a threshold mismatch number of <3. The distances between the start and end positions of the subject sequences are used as the lengths of the matching sequences to obtain N datasets of sequence lengths. Finally, the lengths in each dataset are sorted in descending order of supported read number, and the sorted lengths are numbered in ascending order, resulting in dataset2 with N subsets containing the LNR information. In the third step, the N length distributions in dataset1 are intersected with dataset2, and the lengths with minimum rank differences <3 are retained and labeled as LNR-joint_i_. Then, each LNR-joint_i_ is subjected to another filtration according to the supported read number. To determine the genotype of each LNR-joint_i_, the length with the maximum supported read number is retained if the ratio of the maximum supported read number to the second maximum supported read number is >3; otherwise, the lengths with the maximum and second-maximum supported read number are retained. Finally, N genotypes are obtained. In the fourth step, the N genotypes are combined for statistical analysis, and the results with the mode and supported read number are selected as the final genotype for this target STR locus, that is, if the mode ratio is>=3, it is considered to be homozygous; otherwise, it is considered to be heterozygous. Since interference such as background noise may affect the results, a secondary correction is performed according to the difference in the order of magnitude of the number of reads (Supplementary Material: “Example-1” section).

NanoSTR is freely available as a Perl program and can be used on Linux-based operating system. Before running, users need to install some dependencies. Porechop (version: 0.2.4) (https://github.com/rrwick/Porechop) was used for data preprocessing, NanoPlot (version: 1.38.0) (De Coster et al., 2018) was used for quality control, and BLAST (version: 2.2.23) (Altschul et al., 1990; Camacho et al., 2009) was installed for alignment. Input data can be nanopore sequencing data in *.fastq format. The output of NanoSTR is the typing result of each targeted STR loci. Users only need to set the extension step size d and the bed file of the target STR loci, and the rest of the steps can be analyzed automatically.

### Simulated data

We downloaded 75 forensic makers from STRBase (Supplementary Table S6) (Gettings et al., 2015), and four markers (DYS392, DYS438, DYS448, and DYS635) were used as the simulated target loci. Reference sequences were extracted from the human reference genome hg38 by extension over distances of 1 kb, 10 kb, and 100 kb upstream and downstream of each STR locus. NanoSim-H (version: 1.1.0.4) (Yang et al., 2017) was used to simulate 100,000 nanopore sequencing reads with and without errors based on the extracted sequences (Supplementary Table S1, named Simulated_data-1). Similarly, we simulated heterozygous STR loci with four insertions (Supplementary Table S1, named Simulated_data-2) and four deletions (Supplementary Table S1, named Simulated_data-3) based on the repeat unit of each STR marker.

Ten STR loci (D12S391, D18S51, D22S1045, DYS635, DYS437, DYS438, DYS390, DYS392, DYS448, and DYS458) were randomly selected to assess the effect of the number of errors on genotyping performance. Reference sequence extraction was performed on the human reference genome hg38 with an extension distance of 100 kb upstream and downstream of these STR loci. NanoSim-H (version: 1.1.0.4) was used to simulate 100,000 nanopore sequencing reads with random proportions of mismatches, insertions, and deletions based on the extracted sequences (Supplementary Table S2, named Simulated_data-1). Similarly, we also simulated sequences with four insertions or four deletions based on the repeat unit of each STR marker (Supplementary Table S2, named Simulated_data-2 and Simulated_data-3).

### Experiment with real data

Two genomic DNA standard products, named 2800M (Promega Biotech Co., Ltd., Beijing, China) and 9948 (AGCU ScienTech Incorporation, Wuxi, Jiangsu, China), were used in this study. They contained 51 and 72 Y-STR and/or autosomal STR loci, respectively. Next, we performed two rounds of PCR amplification by using the MultipSeq^®^ Custom Panel (IGMU339V1hg38) kit (iGeneTech Biotech (Beijing) Co., Ltd., Beijing, China) according to the manufacturer’s user guide. Notably, we designed two pairs of primers to replace the amplification primers during the second-round PCR amplification, which were P5-BC02: 5’-(phos). AATGATACGGCGACCACCGAGATCTACACTCGATTCCGTTTGTAGTCGTCTGTACACTCTTTCCCTACACGACGCTCTTCCGATCT-3’, P7-BC12: 5’-(phos)CAAGCAGAAGACGGCATACGAGATCAGGTAGAAAGAAGCAGAATCGGAGTGACTGGAGTTCCTTGGCACCCGAGAATTCCA-3’, P5-BC03: 5’-(phos)AATGATACGGCGACCACCGAGATCTACACGAGTCTTGTGTCCCAGTTACCAGGACACTCTTTCCCTACACGACGCTCTTCCGATCT-3’, and P7-BC13: 5’-(phos) CAAGCAGAAGACGGCATACGAGATAGAACGACTTCCATACTCGTGTGAGTGACTGGAGTTCCTTGGCACCCGAGAATTCCA-3’.That is, after obtaining the first-round PCR products of 2800M and 9948, we used these four specific barcode primers to carry out the second-round PCR amplification. Then, we performed end-repaired and ligated nanopore sequencing adapters to build sequencing libraries. We also performed three experimental replicates for each standard sample. Finally, all sequencing libraries were nanopore-sequenced on the Oxford Nanopore Technology’s MinION (R9.4 chemistry) according to the manufacturer’s instructions, and Guppy (version: 6.1.1+1f6bfa7f8) and model r9.4.1_450bps_hac were used for base calling.

### Data analysis

We used NanoSTR (step size = 10) to analyze the simulated data. We also used NanoSTR (step size = 10) as well as Tandem-Genotypes and TRiCoLOR (version: v1.1) with default parameters (Bolognini et al., 2020) to genotype 44 target STR loci in the standard samples. Minimap2 (version: 2.21-r1071) (Li, 2018) and Last (version: 1250) (Kielbasa et al., 2011) were installed for alignment, and Sambamba (version: 0.8.0) (Tarasov et al., 2015) was installed for alignment processing.

## Results

### Performance on simulated data

**FIGURE 2:**
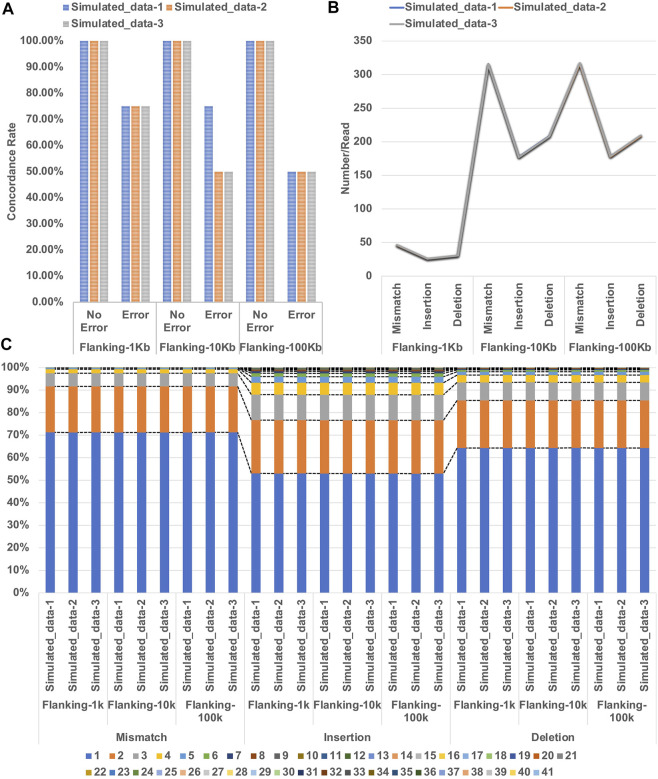
**(A)** Concordance of STR typing with the three simulated datasets of Flanking 1 kb, Flanking-10 kb, and Flanking-100 kb **(B)** error number averaged over reads; and **(C)** proportions of the lengths of each error type.

Analysis of the three error-free simulated datasets (included in Flanking-1Kb, Flanking-10 Kb and Flanking-100 Kb) showed 100% concordance with the expected genotypes (Supplementary Table S1). However, the three simulated datasets of Flanking-1k and the Simulated_data-1 of Flanking-10k with errors showed 75% concordance. A typing error (an allele with one less repeat unit) occurred at DYS392 in the four simulated datasets. The remaining five simulated datasets showed 50% concordance. Except for the Simulated_data-2 of Flanking-100k with typing errors at DYS392 and DYS635, the remaining datasets showed errors at the markers DYS392 and DYS448 (Figure 2A). We averaged the number of mismatches, insertions, and deletions over reads (Figure 2B) and found that the three simulated datasets showed similar results for Flanking-1k, Flanking-10k, and Flanking-100k. We also performed a statistical analysis on the simulated datasets regarding the distribution of lengths with each error type (Figure 2C) and found that most erroneous sequences were 1–2 bp, with slightly higher length diversity of insertions and deletions. However, the same error type but different flanking lengths showed slight variations in length proportions. We therefore infer that the analytical performance of NanoSTR may be greatly affected by the location of the errors given that the relative proportion and distribution of the erroneous sequence lengths were consistent across the three simulated datasets.

### Effect of the number of errors on STR typing accuracy

**FIGURE 3:**
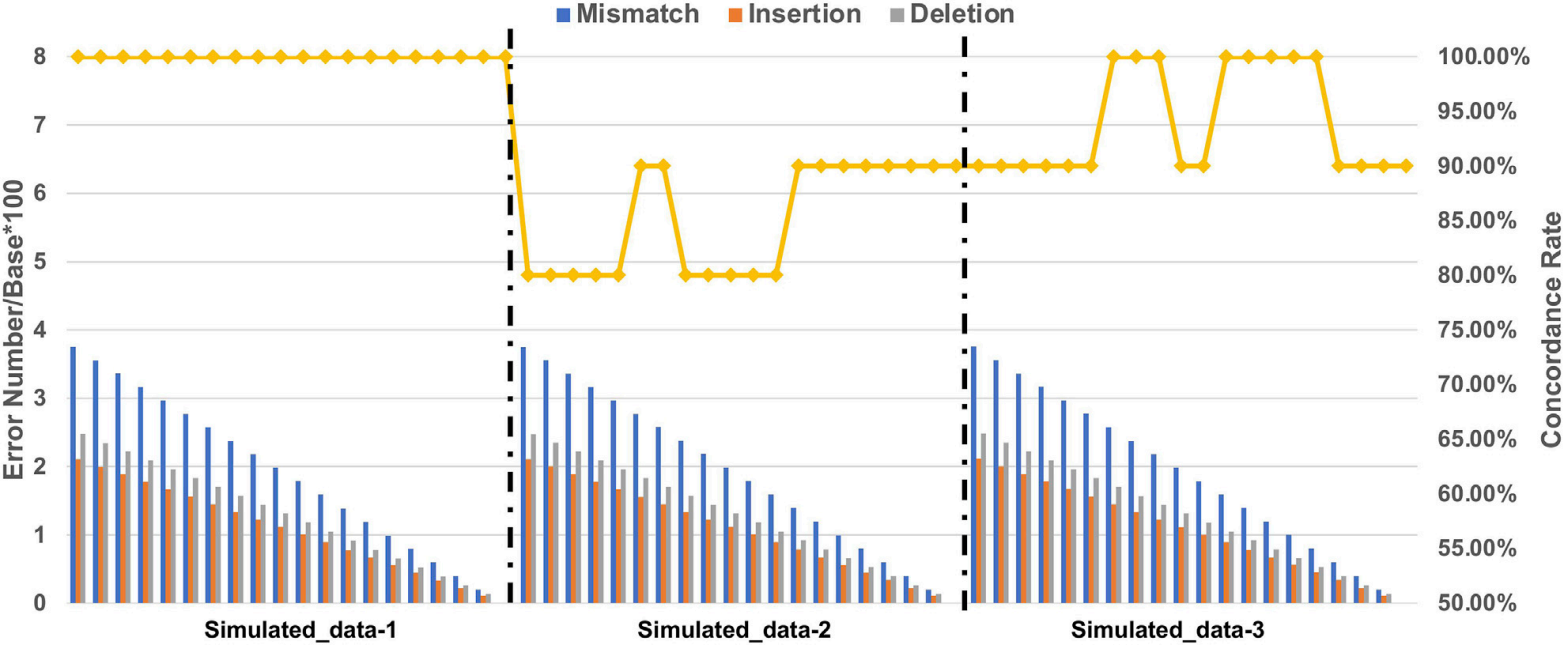
Number of errors and concordance rate of STR typing with simulated datasets. The histograms represent the number of each error type (error number/base*100), and the rhombus symbols connected by a solid line represent the concordance rate with the expected results.

We calculated the ratio of the number of errors/base × 100 of each error type with simulated datasets containing 10 markers (Supplementary Table S2). We found that the accuracy of STR typing decreased with increasing number of errors (Figure 3). Intriguingly, for the Simulated_data-1 with homozygous STR loci, the accuracy remained at 100% regardless of the ratio. For Simulated_data-2 with heterozygous STR loci and an increase of one of the alleles, the accuracy decreased with increasing ratio, and the accuracy was the lowest compared with the other two simulated datasets. For Simulated_data-3 with heterozygous STR loci and one less allele, the accuracy decreased with increasing ratio. We therefore speculate that NanoSTR may perform less well in STR typing for heterozygous loci with increased number of repeats compared to heterozygous loci with reduced number of repeats and homozygous loci in the reference genome. Regarding the performance of NanoSTR, no more than 2.6 mismatches, 1.5 insertions, and 1.7 deletions per 100 bp on average may be necessary to achieve >90% concordance.

### Performance on real data

**FIGURE 4:**
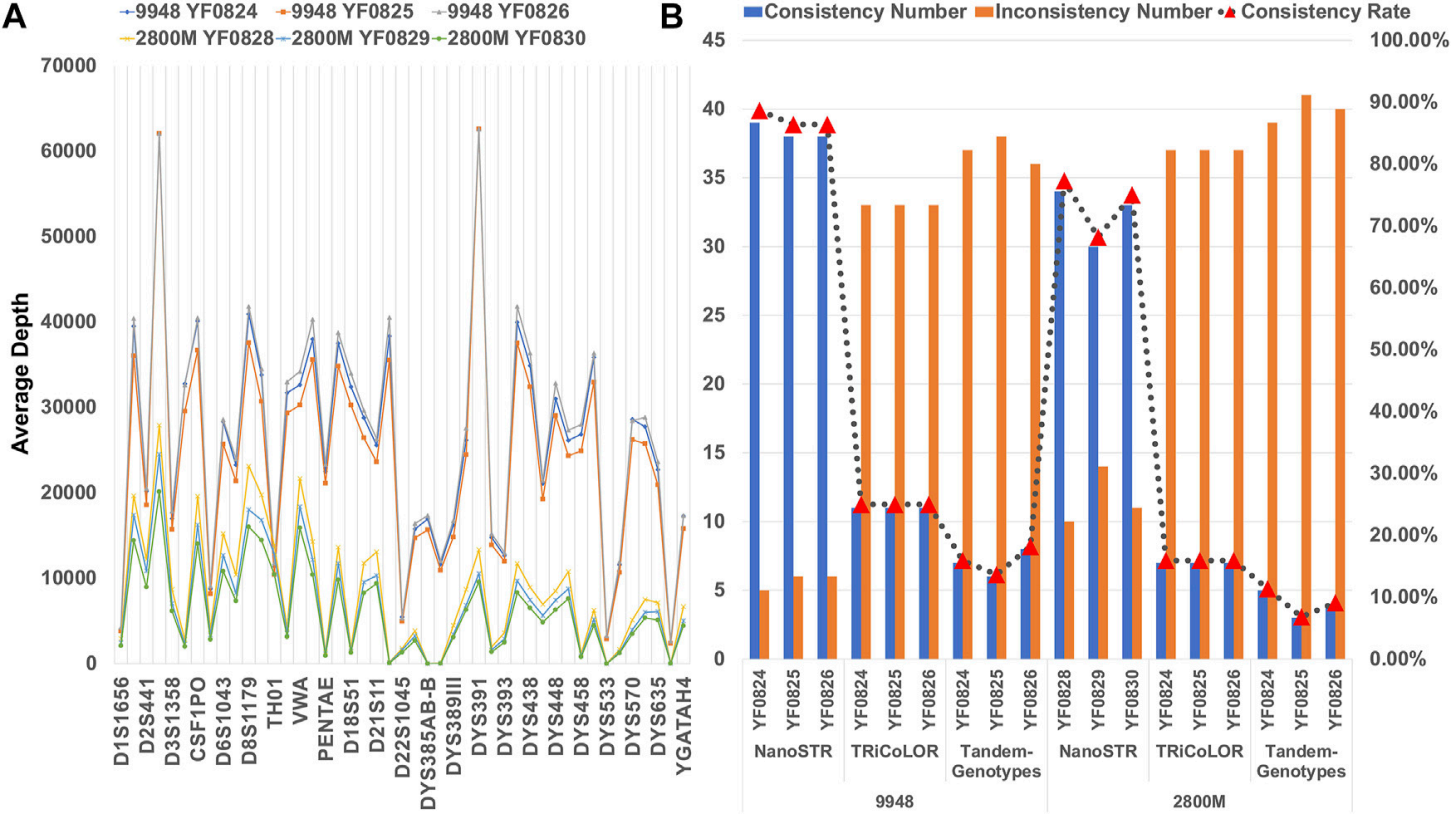
**(A)** Average depth of six standard samples at each STR locus with MinION sequencing platform; **(B)** performance of NanoSTR, TRiCoLOR, and Tandem-Genotypes on the standard samples with Min ION sequencing platform. The bars indicate the number of consistent (blue) and inconsistent (orange) genotypes compared with the standard samples, and the triangle symbols connected by a dotted line represent the concordance rate.

A total of 44 STR loci (DYS385-a/b represents DYS385AB-a and DYS385AB-b) from the intersection of two standard samples (9948 and 2800M) and STRBase with MinION sequencing platform were used for genotype analysis (Supplementary Tables S3, S4). We found similar distributions of average sequencing depth of STR markers in the six control sample datasets (Figure 4A). However, the coverage of some loci was very low in 2800M, which may have affected the genotyping accuracy of some STR markers. We compared the results of STR typing with the standard sample datasets using NanoSTR, Tandem-Genotypes, and TRiCoLOR. We found that NanoSTR showed better analytical performance (Figure 4B). NanoSTR achieved the best performance on 9948 and 2800 M, with 86.36% and 73.48% concordance, respectively. Tandem-Genotypes showed the worst performance; the concordance was only 15.91% and 9.09% for 9948 and 2800 M, respectively. TRiCoLOR showed 25.00% and 15.91% concordance.

## Discussion

Nanopore sequencing, or long-read sequencing, has many advantages over short-read sequencing (Pollard et al., 2018). Compared with Illumina’s commercial short-read sequencing platforms such as HiSeq, NextSeq, and MiSeq, which produce read lengths of up to 600 bp (Bentley et al., 2008), long-read sequencing technologies can generate reads with >10 kb or even >1 Mb base pairs (Wang et al., 2021). However, short-read sequencing has evolved rapidly over the past decade and is highly cost-effective and efficient. It provides sequencing data with high accuracy and has a variety of well-established data analysis tools and workflows (Goodwin et al., 2016). These features are currently lacking in long-read sequencing platforms (Amarasinghe et al., 2020). Due to the highly repetitive and complex structure of STR loci, both next-generation sequencing (NGS) and nanopore-based platforms face some technical challenges in the sequencing, calling, and analysis of STR loci. For example, it is well-known that continuous single-base repeats cannot be accurately sequenced and high-GC and high-repeat regions cannot be efficiently amplified by PCR. Therefore, the accurate detection of STR loci is inherently challenging, and there are particularly urgent and high demands for methods and accuracy of bioinformatics analysis.

NanoSTR is a software for target STR profiling based on long reads from nanopore sequencing. Compared with other analysis methods, NanoSTR can be used to accurately genotype STR loci based on multisampling and LNR of reads. NanoSTR largely circumvents the errors or failure of genotyping associated with nanopore sequencing data characteristics. Moreover, there is no need to establish a genomic background database or align the sequencing data against the human reference genome, thus reducing the consumption of computational resources. There is no requirement for secondary processing steps such as plotting to assist the interpretation of STR genotypes, which saves a considerable amount of time in the analysis. The robustness of NanoSTR is also good, and it can be used on different sequencing platforms and is better than some analysis methods. For example, we also sequenced all libraries using the Qnome-3841 instrument (Qitan Technology (Beijing) Co., Ltd., Beijing, China) according to the manufacturer’s instructions. Then, we performed the NanoSTR analysis process for standards 9948 and 2800M with the Qnome-3841 sequencing platform (Supplementary Tables S3, S5). The results showed the same conclusion with the MinION sequencing platform. That means the similar distributions of average sequencing depth of STR markers in the standard samples (Supplementary Figure S3A) and the best performance of NanoSTR (Supplementary Figure S3B). The concordance rate of NanoSTR on 9948 and 2800M was 71.97% and 53.03%, respectively. Tandem-Genotypes showed the worst performance; the concordance was only 12.88% and 9.85% for 9948 and 2800 M, respectively. TRiCoLOR showed 25.00% and 15.91% concordance. Similarly, it can also be seen that due to differences in different sequencing platforms or experimental steps (Supplementary Figure S2), the performance is slightly different, which also suggests that users need to consider the data characteristics from different sources and need to evaluate and then decide whether the parameters of NanoSTR are even applicable. For both simulated data and real data with MinION and Qnome-3841 sequencing platform, further analysis revealed that the inconsistent genotypes presented by TRiCoLOR and Tandem-Genotypes were completely different. TRiCoLOR showed incorrect STR genotypes whereas Tandem-Genotypes failed to detect some STR loci and produced false negative results. This may be explained by the mechanisms of the algorithms. TRiCoLOR cannot effectively distinguish heterozygous STR loci using datasets without a marked source of haplotypes. Therefore, to some extent, it seems unfair to use our data to evaluate the performance of TRiCoLOR to distinguish heterozygous STR loci. Tandem-Genotypes relies heavily on the accuracy of the genomic background database and alignment algorithm, which may lead to false negative results due to mismatches. These findings explain the limitations and insufficient robustness of TRiCoLOR and Tandem-Genotypes, and further analysis will be performed in our future work to find alternative explanations.

NanoSTR has some limitations and shortcomings. First, this method relies on LNR of reads to detect and genotype STR loci and therefore can be significantly affected by the distribution, size, number, and sequencing depth of random and/or non-random indels. Second, several threshold values are used in this method, such as the rank difference, the ratio of supported read number, and the number of mismatches in BLAST alignment, which may have sizeable impacts on typing performance. For example, the 164-bp DYS389III in the reference genome showed 12 mismatches, and therefore, similar reads were filtered out despite the fulfillment of other criteria. This reduced the number of valid sequences and increased the errors in genotyping (Supplementary Material: the “Example-2” section, Supplementary Figure S1). In contrast, retainment of sequencing reads with excess mismatches can lead to false positive results. Therefore, it is necessary for users to balance these opposing effects according to the data characteristics and actual situations. Third, the method can be limited by the alignment software. BLAST alignment shows the number of gaps, but the length of each gap is unknown, which impedes systematic evaluation of the specific effects of these indels on the typing results. In addition, for STR sites with complex structures, such as [A]n[B]nNn[C]n[D]n, the alignment analysis of BLAST also has challenges, which may easily lead to STR typing errors. Fourth, NanoSTR is not suitable for detection of genome-wide STR loci because it was designed for target STR loci. Fifth, as with other analytical methods and software, NanoSTR is highly dependent on the quality of sequencing data. Theoretically, the higher the accuracy of sequencing, the better would be the performance of NanoSTR. Sixth, some parameter thresholds in the method, such as mismatch number and/or minimum rank differences, were based on the comprehensive evaluation of the sensitivity, specificity and consistency in the simulation data and real data. Users can modify these parameters appropriately according to the actual data characteristics and performance. Therefore, the performance of NanoSTR in the detection of large-size samples requires additional investigation, and more real-world data are needed for further verification.

In summary, NanoSTR still needs further development and optimization in terms of typing accuracy, computational resource consumption, running time, and statistical algorithms. Our results confirm that a single analytical method cannot detect all STR markers. Methods can be used in combination, or some STR loci can be detected by different methods. We will improve the accuracy of STR typing by incorporating deep learning algorithms and electric current distribution in NanoSTR algorithms. We hope that these efforts will increase the performance of NanoSTR and provide a reference bioinformatics analysis method for the application of nanopore sequencing-based STR detection in scientific research and clinical scenarios. As a result, nanopore sequencing technology will be able to truly aid the development of the sequencing industry and the commercialization of precision medicine.

## Conclusion

NanoSTR is a method for STR typing based on nanopore sequencing data and the reads’ length-number-rank information. NanoSTR not only improves the effective use of sequencing data but also shows higher accuracy compared with the existing genotypical methods. NanoSTR provides an alternative analytical method for the detection of STR loci by nanopore sequencing and adds to the related data analysis tools. We hope that NanoSTR can further expand the application of nanopore sequencing techniques in scientific research and clinical scenarios so that these techniques can better promote the development of the sequencing industry and serve the needs of precision medicine.

## Data Availability

The download link of the STRBase database is https://strbaseb.nist.gov/FactSheets/FactSheets_2. FASTQ data files for this study can be found in the NCBI Sequence Read Archive (SRA) database (BioProject ID: PRJNA846950). The codes are available at https://github.com/langjidong/NanoSTR.
