# Supplementary material for: NanoSTR: A method for detection of target short tandem repeats based on nanopore sequencing data
Source: Front Mol Biosci. 2023 Jan 18;10:1093519. doi: 10.3389/fmolb.2023.1093519 (PMC9889824; doi:10.3389/fmolb.2023.1093519)
Supplement: Supplementary file 1 [file DataSheet1.PDF]

- **Example-1:** The example of NanoSTR analysis principle and process

Making the DYS392 site as an example, the parameters of NanoSTR are set to step size=10, and the times of extractions is N=10. The LNR information result format is *LNR-1/2.Times: Number, Length, and (Rank).*

==

Dataset1

LNR-1.1: 375 59 （1）

LNR-1.2: 373 79 （1）

LNR-1.3: 371 99 （1）

30 11097 （2）

LNR-1.4: 371 119 （1）

2 21849 （2）

LNR-1.5: 370 139 （1）

LNR-1.6: 370 159 （1）

5 14484 （2）

LNR-1.7: 370 179 （1）

LNR-1.8: 370 199 （1）

LNR-1.9: 370 219 （1）

3 15606 （2）

LNR-1.10: 370 239 （1）

1 28214 （2）

==

Dataset2

LNR-2.1: 375 59 （1）

LNR-2.2: 373 79 （1）

LNR-2.3: 371 99 （1）

LNR-2.4: 371 119 （1）

LNR-2.5: 370 139 （1）

LNR-2.6: 370 159 （1）

LNR-2.7: 370 179 （1）

LNR-2.8: 370 199 （1）

LNR-2.9: 370 219 （1）

LNR-2.10: 370 239 （1）

==

The 10 length distributions in dataset1 are intersected with dataset2, and the lengths with minimum rank differences < 3 are retained and labeled as LNR-joint$_i$.
LNR-joint-tmp$_1$: 59
LNR-joint-tmp$_2$: 79
LNR-joint-tmp$_3$: 99
LNR-joint-tmp$_4$: 119
LNR-joint-tmp$_5$: 139
LNR-joint-tmp$_6$: 159
LNR-joint-tmp$_7$: 179
LNR-joint-tmp$_8$: 199
LNR-joint-tmp$_9$: 219
LNR-joint-tmp$_{10}$: 239

As the length of the seed sequence of the step size is 10*2, 20*2, 30*2, 40*2, 50*2, 60*2, 70*2, 80*2, 90*2 and 100*2, the lengths of the STR loci are as follows:
LNR-joint$_1$: 59-10*2=39
LNR-joint$_2$: 79-20*2=39
LNR-joint$_3$: 99-30*2=39
LNR-joint$_4$: 119-40*2=39
LNR-joint$_5$: 139-50*2=39
LNR-joint$_6$: 159-60*2=39
LNR-joint$_7$: 179-70*2=39
LNR-joint$_8$: 199-80*2=39
LNR-joint$_9$: 219-90*2=39
LNR-joint$_{10}$: 39-100*2=39

Finally, the 10 genotypes are combined for statistical analysis, and the results with the mode and supported read number are selected as the final genotype for the DYS392 locus, that is, the final type of the DYS392 locus is 39. As the length of the repeat unit

(ATA) is 3-bp, the repeated times is 13 and the STR typing is $[ATA]_{13}$, which represents the homozygous STR loci.

- **Example-2:** DYS389I/II locus typing error analysis

The genomic location of DYS389I/II is chrY: 12,500,448-12,500,611, and the STR sequence on the reference genome (length: 164-bp) is TAGATAGATAGATAGATAGATAGATAGATAGATAGACAGACAGACAGATACATAGATAATACAGATGAGAGTTGGATACAGAAGTAGGTATAATGATAGATAGATAGATAGATAGATAGATAGATAGATAGATAGATAGATAGATAGACAGACAGACAGACAGACAGA, which is [TAGA]n=9 [CAGA]n=3 N48 [TAGA]n=12 [CAGA]n=5。

The Figure S1 showed one cause of error in STR locus typing analysis.

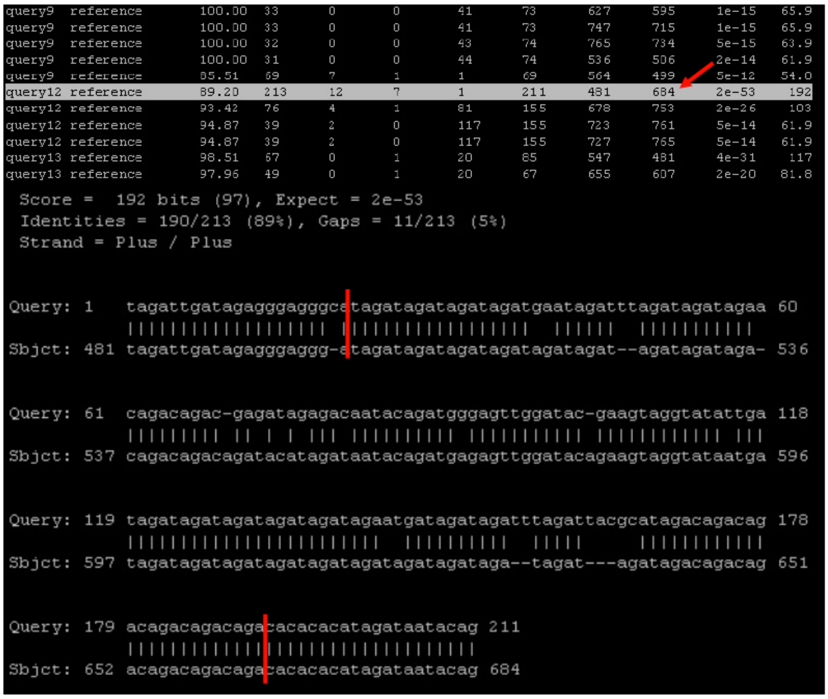

Figure S1. The details of one blast alignment of the DYS389I/II site.

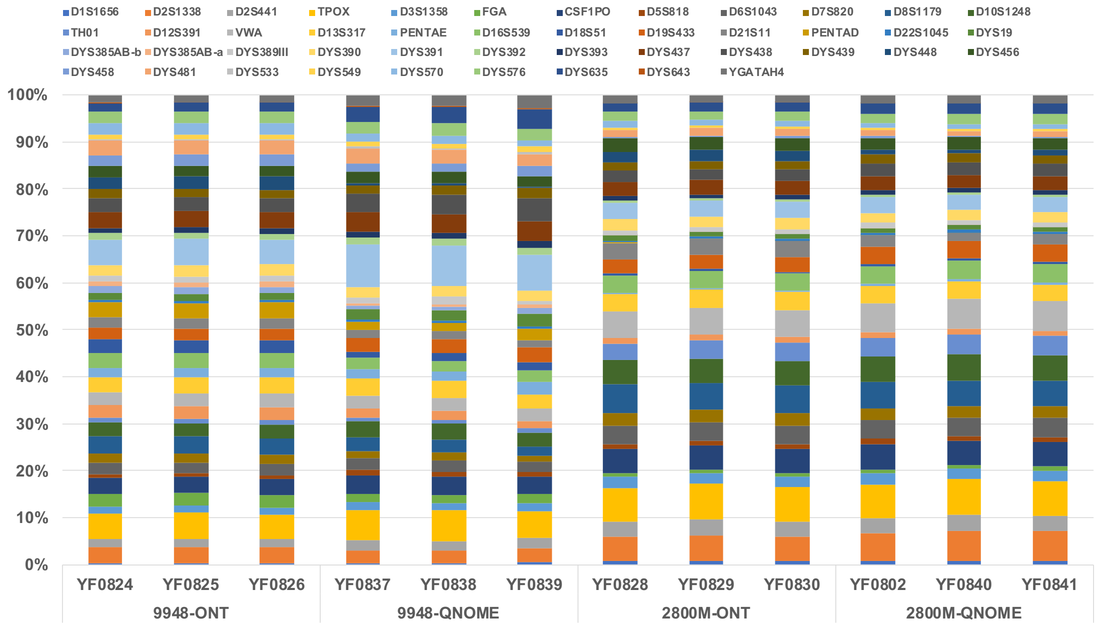

Figure S2. Average depth relative percentage distribution for each amplification interval for standards 9948 and 2800M with MinION and Qnome-3841 sequencing platform, respectively.

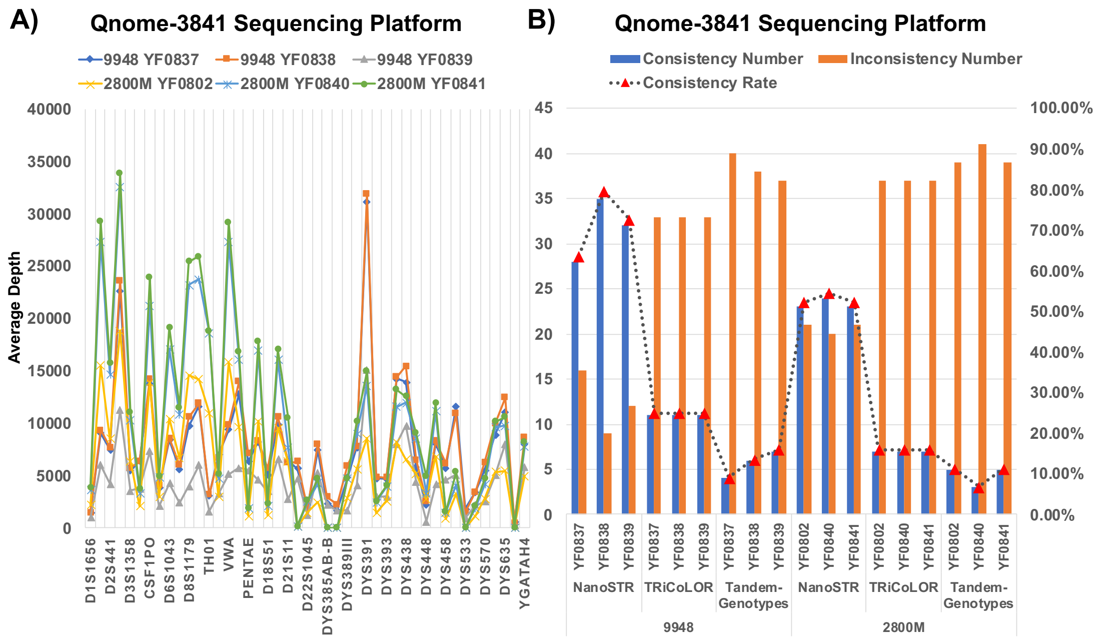

Figure S3. A) Average depth of six standard samples at each STR locus with Qnome-3841 sequencing platform; and B) performance of NanoSTR, TRiCoLOR, and Tandem-Genotypes on the standard samples with Qnome-3841 sequencing platform. The bars indicate the number of consistent (blue) and inconsistent (orange) genotypes compared with the standard samples, and the triangle symbols connected by a dotted line represent the concordance rate.
